# Case Report: Desmoid tumor response to magnetic resonance-guided high intensity focused ultrasound over 4 years

**DOI:** 10.3389/fonc.2023.1124244

**Published:** 2023-06-09

**Authors:** Sin Yuin Yeo, Grischa Bratke, Peter Knöll, Sebastian Gottfried Walter, David Maintz, Holger Grüll

**Affiliations:** ^1^ Institute of Diagnostic and Interventional Radiology, Faculty of Medicine and University Hospital of Cologne, University of Cologne, Cologne, Germany; ^2^ Department of Orthopedic Surgery and Traumatology, Faculty of Medicine and University Hospital of Cologne, University of Cologne, Cologne, Germany; ^3^ Department of Chemistry, Faculty of Mathematics and Natural Sciences, University of Cologne, Cologne, Germany

**Keywords:** HIFU, MR-HIFU, focused ultrasound, magnetic resonance-guided high intensity focused ultrasound, desmoid tumor, tumor control

## Abstract

Desmoid tumors are a rare form of cancer, which show locally aggressive invasion of surrounding tissues and may occur anywhere in the body. Treatment options comprise conservative watch and wait strategies as tumors may show spontaneous regression as well as surgical resection, radiation therapy, nonsteroidal anti-inflammatory drugs (NSAID), chemotherapy, or local thermoablative approaches for progressive disease. The latter comprises cryotherapy, radiofrequency, microwave ablation, or thermal ablation with high intensity focused ultrasound (HIFU) as the only entirely non-invasive option. This report presents a case where a desmoid tumor at the left dorsal humerus was 2 times surgically resected and, after recurrence, thermally ablated with HIFU under magnetic resonance image-guidance (MR-HIFU). In our report, we analyze tumor volume and/or pain score during standard of care (2 years) and after HIFU treatment over a 4-year follow-up period. Results showed MR-HIFU treatment led to complete tumor remission and pain response.

## 1 Introduction

Desmoid fibromatosis, also known as aggressive fibromatosis, desmoid tumor or desmoid-type fibromatosis is a soft tissue tumor characterized by circumscribed proliferation and myofibroblast-like cells (1). It is ranked as an intermediate soft tissue tumor due to the local aggressive behavior but its lacking potential for distant metastases (2). The incidence ranges from 2.4 to 4.3 cases per million inhabitants (3) with most patients diagnosed at the age of 30 years (ranging from 15 to 60 years) (4). Although most of these tumors occur sporadically there is a relevant genetic disposition by familial adenomatous polyposis (FAP) coli, which represents an 800-fold increased risk and is responsible for 7.5% of all desmoid tumors (5). The pathogenesis is not fully understood yet but an association with mutations in the β-catenin pathway for the sporadic patients (6) as well as prior trauma and pregnancy (especially scar after cesarean section) have been described (7). With regards to location, it can be subdivided into abdominal, extra abdominal and lesions of the abdominal wall. Historically, treatment strategies favored surgical resection, however, depending on the complex biological nature as well as exact location of the tumor, high rates for local recurrence were observed. Poor prognostic factors for local recurrence are abdominal and extra abdominal tumors compared to abdominal wall location, age ≤ 25 years and tumor size > 10 cm with a 5-year recurrence-free survival (LRFS) ranging from 90% for abdominal wall tumors to 34% for extremity tumors in patients under 25 years (8). Interestingly, the margin status between R1 and R0 resection only had a significant impact for the subgroup of small tumors (< 5 cm) (8). A detailed analysis of different treatment strategies was recently presented by the Desmoid Tumor Working Group suggesting a conservative watch and wait approach as tumor may show spontaneous regression without any treatment. Only for progressive disease and if symptoms occur, a more active treatment approach should be considered (9). The most common treatment option in this situation is still surgical resection while other potential treatment options include nonsteroidal anti-inflammatory drugs (NSAID), radiation therapy, chemotherapy, or local therapy. Radiation therapy allows for local tumor control in about 80% (10, 11). NSAID´s alone or in combination with anti-hormonal medications like tamoxifen offer the least side effect but with limited response rates. Chemotherapy should only be considered in case of life-threatening and inoperable desmoid fibromatosis (12). While the initially tested tyrosine kinase inhibitor imatinib showed only a progression arrest rate of 45% after 2 years (13) the progression-free survival for sorafenib was 81% after 2 years (14). Cryoablation as a local therapy showed promising first results with progressive disease in 0-4.3% (15, 16). Statistically, more than 50% of patients will have an indolent disease course (17) with spontaneous regression in about 30% for abdominal wall tumors (18). As a high local recurrence rate of 50% was observed after surgical resection, the current tendency for treatment favors more active surveillance and less surgical intervention (19). Consequently, the Desmoid Tumor Working Group recommends active surveillance as the primary approach and medical therapies or individually assessed local ablative treatments as second therapy for all sites but the abdominal wall (9). Recently, the drug nirogacestat, which is selective, small molecule gamma secretase inhibitor showed positive results in the DeFi trial (NCT03785964) for treatment of adult patients with progressing desmoid tumors, which may further impact treatment strategies (20). However, until now, interventional thermoablative therapies such as cryoablation, radiofrequency, microwave ablation or ablation using high intensity focused ultrasound (HIFU) still play a role in local treatment of desmoid tumors (21).

HIFU is a non-invasive, ionizing radiation free, thermal therapy that has received regulatory approvals for treatment of uterine fibroids, bone metastasis, osteoid osteoma, prostate cancer, desmoid tumors and others (22, 23). HIFU can be performed under magnetic resonance imaging- (MR-HIFU) or ultrasound-guidance (US-HIFU), with the earlier approach utilizing real-time temperature mapping and thermal dose information to monitor tissue necrosis during the treatments, while the latter relies on change in echogenicity. Independent of the imaging guidance modality, technical success of ablation or ablation efficiency was in general assessed based on the non-perfused volume (NPV) quantified using post contrast T1-weighted MR images, which is a readout for tissue necrosis. The application of HIFU for desmoid fibromatosis was first demonstrated by Wang et al. in 2011 (24). US-HIFU was used to treat either recurrent or primary extra abdominal desmoids in 10 patients. The treated tumors significantly shrank >50% in volume during a mean follow up of 30 months (24). Thereafter, multiple publications have reported the use of HIFU not only for treatment of extra abdominal desmoids but is also feasible for treatment of intra-abdominal and abdominal wall desmoids (24–34). Desmoid tumors can be treated in single or multiple sessions of HIFU depending on volume but also in case of recurrence. A recent study has shown that there were no significant differences in the incidence of adverse events between desmoids at different anatomical locations, between single and multiple treatments, and between the first and subsequent multiple treatments (25). The tumor reduction rate was reported as mean, median or single cases and varied between 33-100% (24–34), and the 5-year estimated progression-free survival rate in a single study of 91 patients treated with US-HIFU was 69.3% (34). Previous publications have described a tumor reduction rate based on the final follow-up time point for each patient, leading to highly variable treatment efficacy at different time points (24–34). Also, the number of patients experiencing complete response remained small and the contributing factors leading to tumor remission have not been discussed. Besides that, the feasibility of MR-HIFU to provide pain relief has only been reported in a small number of patients, up to 8 months follow-up (27, 29). Therefore, we would like to complement current literature with a case report were we assessed the desmoid tumor reduction rates at multiple time points over 4 years after MR-HIFU treatment, as well as the efficacy of MR-HIFU ablation for long-term pain management.

## 2 Case presentation

This retrospective analysis has been approved by the local institutional review board, and informed consent for the MR-HIFU therapy has been obtained from the patient. We analyzed a 66-year-old, female patient with desmoid tumor at the left dorsal humerus, who has been treated at our institution between January 2016 to January 2018 and followed up for 4-years. The patient was first diagnosed with a beta-catenin positive desmoid tumor but without CTNNB1 mutation in January 2016, which was surgically (R0-resection) removed in March 2016. During the follow up in February 2017, recurrent growth was observed. In March 2017, a second R0-resection was performed. Thereafter, a COX-2 inhibitor was prescribed for a period of 9 months between March to November 2017. In January 2018, desmoid tumor recurred again. Following tumor board discussion, the patient first underwent ulnar nerve repositioning moving it medially towards the subcutaneous fatty tissue to spare it from any potential damage during HIFU ablation. Subsequently, MR-HIFU ablation was performed as an alternative treatment to surgery.

**Figure 1:**
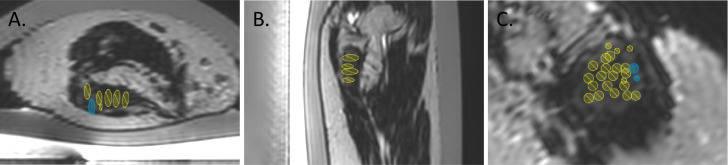
Representative T2-weighted treatment planning images in axial **(A)**, sagittal **(B)** and coronal **(C)** views showing placements of MR-HIFU treatment cells (ellipsoids).

**Figure 2:**
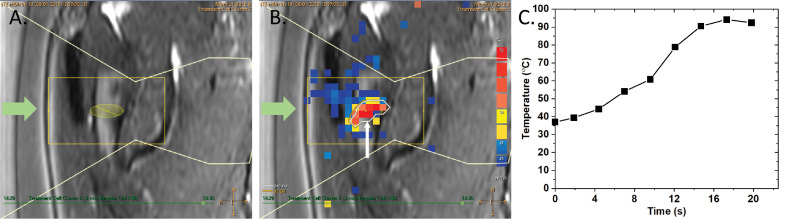
Representative example of a MR-HIFU ablation using a 4 mm treatment cell, 1.2 MHz, 60 W sonication power and 14.9 s sonication duration. The green arrows **(A, B)** depict the direction of incoming ultrasound energy. **(A)** A treatment cell (ellipsoid) positioned on the desmoid tumor. **(B)** Temperature map of a sonication. White contour and arrow mark the 240CEM43 thermal dose. **(C)** The corresponding temperature increase over time within the treatment cell during a sonication.

Prior to the treatment, the left dorsal humerus was depilated. Then, the patient was placed under general anesthesia and transferred onto a 3 T MR-HIFU treatment platform (Sonalleve®, Profound Medical Inc., Canada). The patient was placed on her left side with the desmoid tumor centered on the transducer. Acoustic contact between the transducer and desmoid tumor was established with degassed water, ultrasound gel (Aquasonic 100, Parker Laboratories, Fairfield, USA), and a gel pad of 1.5 cm in thickness (Aquaflex®, Profound Medical Inc., Canada). Pre-treatment planning images, such as T1-weighted [repetition time (TR) = 3.9 ms, echo time (TE) = 2.0 ms, field of view (FOV) = 200 × 300 × 160 mm^3^, voxel size = 1 × 1 × 2 mm^3^, number of signal averages (NSA) = 2], and T2 turbo spin echo (TSE) mDixon (TR = 3071 ms, TE = 85 ms, FOV = 330 × 400 × 198 mm^3^, voxel size = 0.75 × 0.92 × 5.00 mm^3^, NSA = 1) sequences, were acquired. A total of 34 treatment cells (4 x 4 x 10 mm3) were planned on the desmoid tumor (**Figure 1**). Thereafter, 12 sub-therapeutic sonications (acoustic frequency = 1.2 MHz, acoustic power = 30 W, duration = 4.6 – 15.9 s per sonication) were performed to adjust and confirm focal heating at planned target sites. During sonication, the proton resonance frequency shift (PRFS) thermometry sequence (RF-spoiled gradient with echo planar imaging (EPI) readout, EPI factor = 11, TR = 25 ms, TE = 16 ms, FOV = 400 x 300 mm^2^, voxel size= 2.1 × 2.1 × 7.00 mm^3^, flip angle = 16°, NSA = 1, dynamic scan time = 2.6 s) was used to monitor temperature increase at the focal spot. MR-HIFU ablation was defined as a temperature increase of >60°C. The 240 cumulative equivalent minutes at 43°C (240CEM43) thermal dose concept was used to determine tissue necrosis (35). **Figure 2** shows a representative example of an ablation. For MR-HIFU therapy, ablations were performed using 1.2 MHz, 40 – 60 W, and 4.2 – 15.9 s sonication duration. At the end of the therapy, a gadolinium-based contrast agent (0.1 mmol/kg body weight, Dotagraf®, Jenapharm GmbH & Co. KG, Jena, Germany) was injected and contrast-enhanced (CE) T1-weighted MR images were acquired to assess NPV. Patient was followed up at 1.5, 3, 10, 23, 35, and 48 months post MR-HIFU treatments. Pain relief was assessed using the visual analog scale (VAS) score, while tumor volume before and after MR-HIFU was evaluated with CE-T1-weighted MRI except for the time point of first diagnosis where a CT image was used. Tumor volume and NPV were quantified using the Horos image analysis software (Nimble Co LLC d/b/a Purview, Annapolis, US). NPV ratio was defined as the ratio (%) of NPV-to-desmoid volume. Treatment-related adverse events were monitored. The whole therapy duration was 3 hours and 35 mins, which could be further sub-categorized into the following workflow components – (i) transfer of patient into the MRI room and positioning = 36 mins; (ii) MR imaging and treatment planning = 41 mins; (iii) HIFU treatment = 108 mins (active sonication = 9 mins; cooling between sonications and treatment optimization = 99 mins); and (iv) post treatment MR imaging = 30 mins).

**Figure 3:**
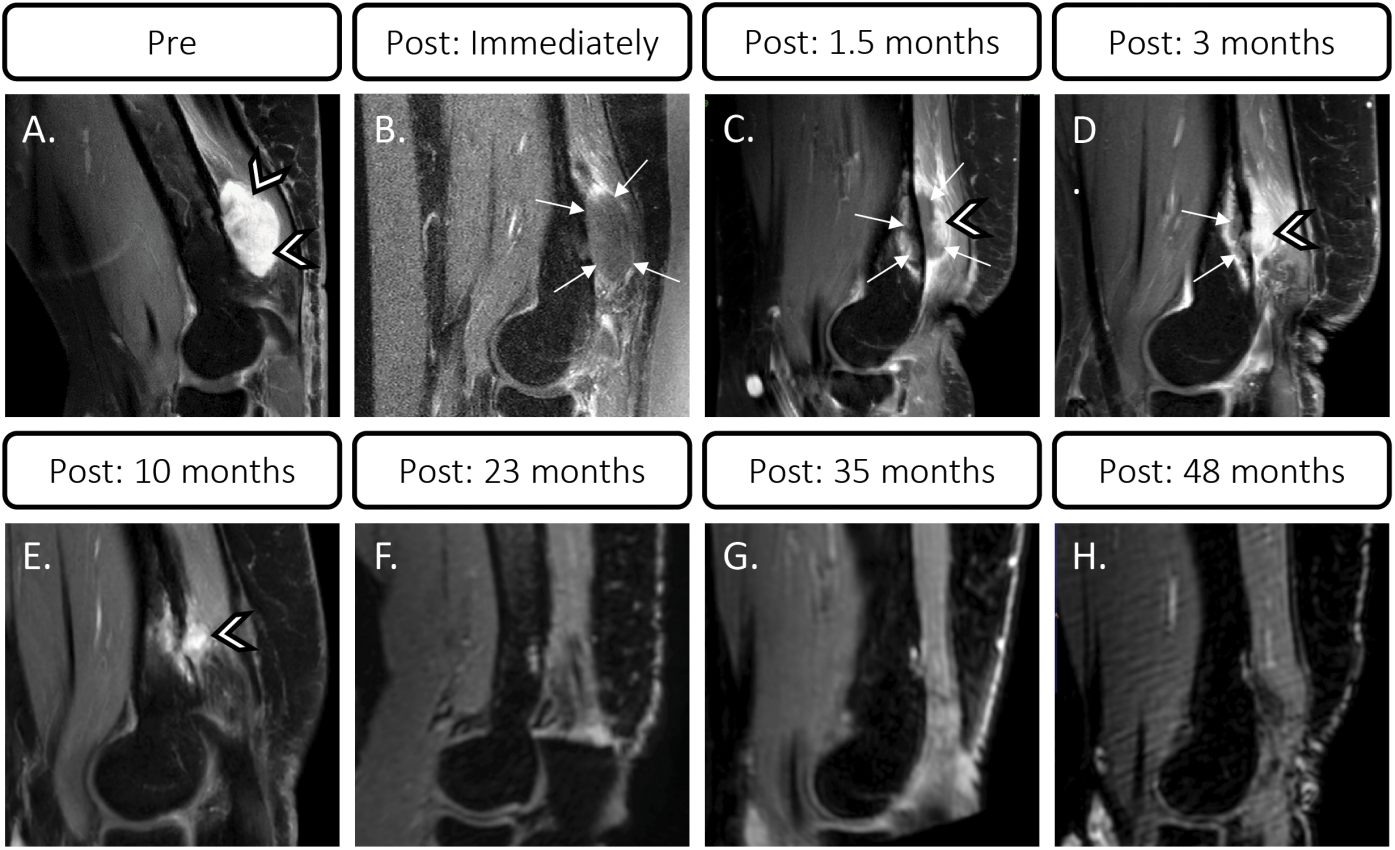
Contrast-enhanced T1-weighted MR images of desmoid tumor before and after MR-HIFU treatment. **(A)** Hyperintense desmoid tumor at dorsal humerus (arrow heads) before MR-HIFU. **(B)** Non-enhancing ablation zone immediately after treatment (arrows) with NPV ratio of 95%. **(C, D)** Tumor (arrow heads) at follow-up showing NPV (arrows) with hyperemic rim at 1.5 and 3 months after treatment. **(E)** Tumor at 10 months post MR-HIFU. **(F)** Complete resolution of desmoid tumor at 23 months post MR-HIFU. No tumor recurrence at 35 **(G)** and 48 months **(H)** after treatment.

**Figure 4:**
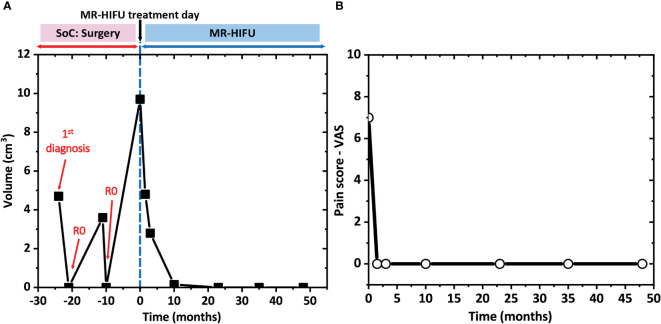
**(A)** Desmoid tumor response over 6 years. After the first diagnosis, the patient underwent 2 times R0 resections, which were standard of care (SoC) treatments, over a period of 2 years. As the tumor recurred after the second resection, the patient was subjected to MR-HIFU treatment and thereafter, was followed-up for 4 years. Time point 0 corresponds to the day of MR-HIFU treatment. **(B)** Pain relief assessed with visual analog scale (VAS) score at different time points after MR-HIFU treatments for 4 years.

As illustrated in **Figures 3A**, **4A**, before MR-HIFU ablation, an T1-weighted CE images showed a desmoid tumor with a volume of 9.7 cm^3^ was noted at the dorsal humerus. Immediately after treatment, a non-enhancing ablation zone was observed with a NPV ratio of 95% (**Figures 3B**). At 1.5 months post MR-HIFU, tumor regression was observed (4.8 cm^3^, 50.5% volume reduction, **Figures 3C**, **4A**). Also, NPV with a hyperemic rim was evident within the desmoid tumor as well as the adjacent bone marrow (**Figures 3C–F**). The tumor continued to shrink to 2.8 (71.1% volume reduction) and 0.15 cm^3^ (98.5% volume reduction) at 3- and 12-month follow-up time points, respectively (**Figures 3D, E**, **4A**). Interestingly, 24 months after MR-HIFU, no tumor was detected, achieving complete response (**Figure 3F**). No tumor recurrence was observed at 35 (**Figure 3G**) and 48 months (**Figure 3H**) after the treatment. **Figure 4A** shows the timeline of tumor response to surgery, which was the standard treatment, and after HIFU. At first diagnosis (-24 months, before MR-HIFU), the tumor had a volume of 4.7 cm^3^. Though the tumor was surgically removed, the tumor regrew to a volume of 3.6 cm^3^ (-11 months, before MR-HIFU). However, a second R0-resection in combination with a COX-2 inhibitor was not successful in controlling tumor growth. Tumor recurrence was observed at 11 months after the second R0-resection and with a tumor volume 1.7 times larger (9.7 cm^3^). As opposed to standard treatments, MR-HIFU provided improved tumor control. After MR-HIFU, tumor reduction was observed over time until complete remission was observed 24 months after treatment with no recurrence up to 48 months (**Figure 4A**). Prior to MR-HIFU treatment, the patient experienced pain with a VAS score of 7. At 1.5 months after MR-HIFU, complete pain relief was observed (**Figure 4B**). The patient remained symptom-free (VAS score = 0) at 3-, 10-, 23-, 35-, and 48-month follow-up time points (**Figure 4B**). No treatment-related adverse events were observed at all-time points after MR-HIFU treatment.

## 3 Discussion

Treatment of desmoid tumors remains a challenge. Based on the evidence-based, joint global consensus guideline approach for management of desmoid tumor, “active surveillance” for 1-2 years is the first step after diagnosis. Thereafter, in case of progression, depending on the anatomical location, such as abdominal wall, intra-abdominal, retroperitoneal, pelvic, extremity, girdles, chest wall, head and neck or intra-thoracic, different treatment options, for examples, surgery, medical treatment, radiotherapy, and/or isolated limb perfusion, can be prescribed in a stepwise approach. However, when these treatment options fail, patients can be offered alternative treatments (9). MR-HIFU is an attractive alternative thermal treatment option due to its non-invasive and ionizing-free nature.

In this case report, we showed that following MR-HIFU treatment, the desmoid tumor volumes reduced by 50.5%, 71.1%, 98.5% and 100%, at 1.5, 3, 10 and 23 months, respectively, with no recurrence at 35- and 48- months follow-up time points. Prior studies, independent of the type of imaging guidance used during treatment, have shown tumor reduction varying between 33 – 100% with a follow-up time point between 3 – 114 months (24–34). Zhang et al. reported a tumor volume reduction rate of 36.1 ± 4.2% at 3 months after US-HIFU treatments in 111 patients (25). In a separate study where 7 patients with intra-abdominal desmoid tumors were treated with US-HIFU, the tumor regression rates were 34.8 ± 8.2% and 58.2 ± 12.7%, at 6 and 12 months after treatments, respectively (33). At 18.2 months follow-up, an average tumor reduction of 36% was observed in MR-HIFU treated patients (28). A mean reduction of 59% in tumor volume was noted at a mean follow-up of 29 months (32). Compared to these results, our data showed a higher percentage of tumor reduction at different follow-up time points.

To date, 19 cases of complete response have been described, of which 5 cases were treated under MR guidance, while the remaining 14 cases were treated under US guidance (27, 30–32, 34). These cases were analyzed in detail in terms of desmoid locations, tumor volume before HIFU treatments, if patients received other treatments prior to HIFU, number of HIFU treatments, NPV ratio after treatments, and follow-up durations. Following analysis, the required information was only available for 6 patients (5 treated with MR-HIFU and 1 treated with US-HIFU) (27, 30, 32). The desmoid tumors were located at the anterior shoulder (n = 1), abdominal wall (n = 3), intercostal muscle (n = 1), and mesentery (n = 1). Five tumors had prior treatments, such as surgery, radiation therapy or cryotherapy, while 1 tumor did not receive any prior treatment. These tumors had an average volume of 13.1 ± 11.1 cm^3^ (range, 3 – 30 cm^3^) and received between 1 – 4 times HIFU treatments. The average NPV ratio was 85.2 ± 22.1% (range 43.3 – 100%) after the treatments. Patients were follow-up for 30.8 ± 17.4 months (range, 9 – 60 months) on average. The patient in this study had comparable characteristics, where a recurrent desmoid tumor, with a volume of 9.7 cm^3^, was treated in a single MR-HIFU session, achieving an NPV ratio of 95% immediately post treatment and complete response starting at 23 months follow-up time point with no recurrence at the 2 subsequent follow-ups at 35 and 48 months. Taken together, current data suggests that initial tumor volume and NPV ratios are potential key factors to achieve complete response with HIFU. In addition, a recommended follow-up period of at least 2 years might be needed to assess tumor response.

Besides tumor control, complete and durable pain relief up to 4 years were noted in this study. This is the longest observation of pain control in comparison to previously published results (27, 29). Ghanouni et al. observed significant pain relief in 6 out of the 15 treated patients. The worst and average daily numerical rating scale (NRS) pain scores reduced from 7.5 ± 1.9 to 2.7 ± 2.6, and 6.0 ± 2.3 to 1.3 ± 2.0, respectively, at a median follow-up of 8 months (4 – 17 months) after treatment. In addition, within 2 weeks after the treatments, all patients discontinued their scheduled pain control medications (27). Another study reported a reduction of pain score from 5 to 0 within 1 month after the HIFU treatment in 1 patient (29). This is in line with our observation where complete pain relief was already observed at 1.5 months after MR-HIFU and all-time beyond.

Despite the limitation of being a single case report, our results not only corroborated the use MR-HIFU for treatment of recurrent desmoid tumors, but also provided an indication of essential criteria for achieving complete remission. A higher number of patients will be needed to provide statistical evidence for the role of MR-HIFU as a treatment option for desmoid tumors. In addition, future studies should focus on understanding criteria needed for successful treatment of desmoid tumors, including, but not limited to imaging characteristics, pathology, HIFU-treatment parameters, etc., relapse-free survival, progression-free survival, role of MR-HIFU for curative as well as palliative intent to maximize the clinical outcomes for patients.

## 4 Conclusion

MR-HIFU is a promising and effective thermal therapy for desmoid tumor eradication and long-term pain relief. Small tumor volume (< 30 cm3) and high NPV ratio (> 85%) are 2 potential essential criteria for complete response, necessitating further investigations. Moreover, a long-term follow-up of at least 2 years may be needed to effectively assess tumor response.

## Data availability statement

The original contributions presented in the study are included in the article/supplementary material. Further inquiries can be directed to the corresponding author.

## Ethics statement

The studies involving human participants were reviewed and approved by University Hospital of Cologne, Germany. The patient signed informed consent to receive an MR-HIFU treatment and written informed consent was obtained for the publication of this case report.

## Author contributions

Conceptualization and methodology: SY, GB, and HG. Data acquisition, analysis, and interpretation: SY, GB, and HG. Writing – original draft preparation: SY, GB, and HG. Writing – review and editing: SY, GB, PK, SW, DM, and HG. Visualization: SY. Funding acquisition: HG. All authors contributed to the article and approved the submitted version.
